# Impact of reflection writing on the learning ability of Indian medical students

**DOI:** 10.6026/973206300200587

**Published:** 2024-05-31

**Authors:** Rekha Jiwane, Vivekanand Gajbhiye, Sandip Hulke, Ruchi Singh, Ragini Shrivastava, Varun Malhotra

**Affiliations:** 1 Department of Physiology, AIIMS, Bhopal, India; 2 Department of Anatomy, RKDF Medical Collage, Bhopal, India

**Keywords:** Reflective writing, physiology, First year students, oedema, body fluids

## Abstract

Reflective writing develops meta-cognition among students. Therefore, it is of interest to compare effectiveness of post lecture reflective writing to didactic lecture between individual and group reflective writing. Hence, we included 124 first-year students from AIIMS Bhopal, India and divided them in two groups of 62 students. Both groups took a pre-test using a reflection questionnaire. Students were taught reflective writing. Both groups attended physiology lectures on two different topics. First lecture on body fluids where Group A wrote reflections individually and Group B did so in sub-groups (B1 to B6). After another lecture on Pathophysiology of oedema, Group A wrote reflections in groups and Group B wrote individually (A1 to A6). Both groups took a test in the form of MCQ about reflective writing on lectures. After intervention both groups took a post-test using a reflection questionnaire. Mean and standard deviation of Pre-test is 3.86 ± 0.86 and Post-test is 7.58 ± 1.01, respectively. The Mean and standard deviation of reflection who wrote individually is 38.05 ± 4.41 and in group is 27.45 ± 3.93, respectively with p-value < 0.05. Evaluation of students who wrote reflection in groups after second lecture the mean and standard deviation of reflection who wrote individually is 38.22 ± 4.64 and in group is 27.03 ± 2.87 respectively with p-value < 0.05. The performance of students who wrote reflection in groups is not satisfactory as compared to students who wrote their reflection individually.

## Background:

Reflective writing is an ability of an individual to reflect on actions, thoughts, and incidents so they are in the process of continuous learning [1]. Experience alone is not sufficient; deliberate reflection on experience is necessary for deep understanding and learning [2, 3]. For teacher education and advancement programs, reflective writing is an important part. To enhance learning, addition of reflection increases clinical thinking and decision-making ability. When students are writing reflections, they are thinking about their work-how, when, and where to do work, trying hard to fulfil required criteria, analyzing the effectiveness of their efforts, and preparing plans for improvement. Reflection is linked to elements that are fundamental to meaningful learning and cognitive development [4]. Reflective practice develops metacognition among students. Metacognition is the ability of students to improve their thinking, self-evaluation, and judgments of the quality of work based on evidence. Reflective writing also develops the ability of critical thinking, problem-solving, decision-making, and enhances understanding of teachers' instructions to students. Reflective practice can be a way of developing autonomous and self-directed learning, which helps students become lifelong learners. Medical professionals and students can combine reflective practice with checklists to reduce diagnostic errors in patient care. It can be incorporated into UG, PG, and CME programs [5]. Therefore, it is of interest to compare the effectiveness of post-lecture reflective writing to didactic lecture between individual and group reflective writing.

## Material and Methods:

## Study design:

This cross-sectional study was conducted in the Department of Physiology at AIIMS, Bhopal, India, from January 2023 to April 2023.

## Participants:

The study involved 124 first-year medical students.

## Ethical considerations:

The study was conducted after obtaining approval from the Departmental Research Committee and ethical clearance from the Research Review Board at AIIMS Bhopal (Approval number: AIIMS/BPL/RRB/Approval/2022/25). All students were informed about the aims and objectives of the study and participated voluntarily.

## Intervention:

## Preparation and counselling:

Before the study commenced, all students received counselling regarding the importance and utility of reflective writing in their academic careers. All students participated voluntarily in the study.

## Group assignment:

Students were divided into two groups, A and B, each comprising 62 students selected according to their roll numbers.

## Training:

Both groups were instructed on how to perform reflective writing individually using a pre-validated reflective writing framework for academic reflection.

## Pre-test:

Prior to the intervention, all students completed a pre-validated questionnaire assessing their understanding of reflection.

## Educational sessions:

Both groups attended interactive physiology lectures on two pre-selected topics related to general physiology, each with defined learning objectives.

## Reflective writing sessions:

## First lecture: 

A lecture on "The Body Fluid" was delivered.

1. After the lecture, Group A students wrote individual reflections.

2. Group B was divided into six sub-groups (B1 to B6) to discuss the lecture content and write reflections in groups.

## Second lecture:

A lecture on "The Pathophysiology of Edema" was delivered.

1. Group B attended the lecture and wrote individual reflections.

2. Group A wrote reflections in groups (A1 to A6) after attending the lecture.

## Post-test:

The day after each lecture, both groups underwent a post-teaching/learning activity test consisting of multiple-choice questions to evaluate their learning outcomes.

## Outcome measures:

Blinded copies of reflective writing papers were collected for analysis. Pre- and post-test outcomes were compared between both groups for each educational encounter was competed.

## Data analysis:

Data analysis of pre-post-test through paired and unpaired t-test grading of reflective writing analysis of student feedback questionnaire was also completed for 64 group A & group B students.

## Pre and post-test reflective writing questionaries' used.

1. What is Reflective Teaching and Learning?

2. What do you mean by reflection writing?

3. Name any two Models of reflective teaching and learning.

4. What are Benefits of Reflective Teaching and Learning?

5. Who get benefited by reflective teaching and learning?

6. What are different components of reflective learning cycles to structure your writing?

7. Did you give your best effort on this most recent assignment?

8. What are some things you really want to do well on this Teaching and Learning process?

9. Will reflective writing help in improving skills and problem-solving abilities of students?

10. Does reflective writing affect performance of students?

11. If you could do this assignment over, what would you do differently?

12. What class activities or assignments help you learn the most?

13. What do you think writing reflection or doing any assignment in group is better than doing it individually?

14. What are some problems you see in the students that you believe that they should work on those problems.

15. What are some of the solutions to those problems?

## Reflection questions to improve learning (dialectic lecture of General physiology)

1. What seem to be most important topic in today's lecture?

2. Briefly describe what you learn in today's lecture?

3. What seem to be least important topic in today's lecture?

4. What was your role in the learning process today? Was it active or passive?

5. When where you at best today?

6. How do you known that you understand?

7. Were teacher covers all subtopics in his/her lecture?

8. What challenges did you encounter? How did you respond?

9. What did you already know that you can use to think about or learn?

10. Did this activity help you learn more than others we've done? Why?

11. Did you come to class today prepared to learn (in both your attitude and with all your supplies)?

## Results:

## Sample size distribution for pre-test and post-test among 124 students:

When we compare mean pre-test and post-test scores of Group A and Group B. (maximum marks = 10) Students have less knowledge about reflection writing. When we explain them about reflection writing and they implement it. They found reflection writing is useful for their further academic performance. Students who are writing reflection in groups is evaluated after lecture performance of these students are not satisfactory as students who wrote their reflection individually.

## Comparison of mean pre-test and post-test scores:

The maximum marks are 10. Students initially had limited knowledge about reflective writing. After being instructed on reflective writing and implementing it, they found it useful for their further academic performance. Students who wrote reflections in groups had less satisfactory post-lecture performance compared to those who wrote reflections individually. The sample size distribution is pre-test and post-test included 124 students. When comparing the mean pre-test and post-test scores of Group A and Group B (maximum marks = 10), it was found that students initially had limited knowledge about reflective writing. However, after being taught about reflective writing and implementing it, they found it useful for their academic performance. Students who wrote reflections in groups showed less satisfactory performance compared to those who wrote their reflections individually.

## Sample size distribution:

The sample size for the pre-test and post-test was 124 students each (Table 1).

## Comparison of pre-test and post-test scores:

The mean and standard deviation for the pre-test were 3.86 ± 0.86, while for the post-test; they were 7.58 ± 1.01. The 95% confidence interval for the pre-test was 3.70 to 4.01, and for the post-test, it was 7.40 to 7.75. The mean difference between the pre-test and post-test scores was -3.72. The t-test value was 36.93, with a p-value < 0.001 indicating statistically significant differences between the pre-test and post-test scores (Table 2).

## Comparison of individual vs. group reflections:

The mean and standard deviation for individual reflections were 38.05 ± 4.41, while for group reflections, they were 27.45 ± 3.93. The 95% confidence interval for individual reflections was 36.93 to 39.17, and for group reflections, it was 26.45 to 28.45. The mean difference between individual and group reflections was 10.59. The t-test value was 15.21, with a p-value < 0.001, indicating statistically significant differences between individual and group reflections (Table 3).

## Repeated comparison of group vs. individual reflections:

The mean and standard deviation for individual reflections were 38.22 ± 4.64, while for group reflections, they were 27.03 ± 2.87. The 95% confidence interval for individual reflections was 37.05 to 39.39, and for group reflections, it was 26.31 to 27.76. The mean difference between group and individual reflections was -11.19. The t-test value was 15.44, with a p-value < 0.001, indicating statistically significant differences between group and individual reflections (Table 4). In the present study, the Mean and Standard deviation of Pre-test is 3.86 ± 0.86 and Post-test is 7.58 ± 1.01 respectively with lower limit of 3.70 & 7.40 (In Pre-test and Post-test respectively) and upper limit of 4.01 & 7.75 (In Pre-test and Post-test respectively) with the 95% confidence interval. The mean difference between the mean values of Pre-test and Post-test is -3.72. With reference to the t-test value and p-value < 0.05 it can be seen that the values are statistically significant between the Pre-test and Post-test. In the present study, the Mean and Standard deviation of Individually they wrote reflection is 38.05 ± 4.41 and in group they wrote reflection is 27.45 ± 3.93 respectively with lower limit of 36.93 & 26.17 (In individual and in group respectively) and upper limit of 39.17 & 28.45 (in individual and in group respectively) with the 95% confidence interval. The mean difference between the mean values in individual and in group respectively is 10.59. With reference to the t-test value and p-value < 0.05 it can be seen that the values are statistically significant between the in individual and in group respectively. In the present study, the Mean and Standard deviation of Individually they wrote reflection is 38.22 ± 4.64 and in group they wrote reflection is 27.03 ± 2.87 respectively with lower limit of 37.05 & 26.31 (In group respectively and in individual) and upper limit of 39.39 & 27.76 (In group respectively and in individual) with the 95% confidence interval. The mean difference between the mean values in group respectively and in individual is -11.19. With reference to the t-test value and p-value < 0.05 it can be seen that the values are statistically significant between the in individual and in group respectively.

## Discussion:

Reflection within undergraduate medical education enhances students self-monitoring of their learning process. It allows students to recognize past mistakes and strategize on how to improve, thereby optimizing their learning opportunities for personal development. Serving as a tool for lifelong learning, reflection facilitates continuous growth [6]. The findings of our study align with core components essential for meaningful learning and cognitive development throughout a student's professional journey. It empowers students to enhance their thought were observed among the different professional groups, notable differences emerged between undergraduate and postgraduate students across all four constructs; postgraduates exhibited a higher tendency towards employing deeper forms of reflection. The study involved participation from undergraduate and postgraduate students (totalling 303) in fields such as occupational therapy, physiotherapy, radiography, and nursing. Across all groups, habitual action and critical reflection-representing the least and most analytical levels, respectively-were found to be the least frequently demonstrated. There is no statistically significant variance in reflective, critical thinking self-assessment, and judgment of work quality based on evidence. Developing such skills and attitudes in students is crucial not only for their professional advancement but also for their holistic growth. A contemporary medical education and practice, there is a growing need for innovative approaches to enhance student learning. Reflective practice stands out as a well-established tool for improving learning outcomes after teaching sessions. It involves an analytical approach where individuals incorporate personal reflections on actions, incidents, situations, or thoughts [7]. Levine and colleagues investigated the effects of reflective writing on learning enhancement among residents. They found that residents exhibited greater self-awareness and a re-evaluation of their fundamental beliefs when engaging in reflection. The authors suggested that reflection played several roles for residents, including providing an outlet for emotional expression, facilitating the clarification of learning objectives, and serving as a source of motivation for self-improvement [8]. Stuart *et al.* (2020) conducted a study where they analyzed the reflective writing of senior medical students participating in a coordinated reflection education program during their clerkships in General Practice, Pediatric, and Psychiatry. They examined 135 reflection assignments and found common themes across the three clerkships, particularly related to students' emotional struggles in developing a professional identity. Specifically, students on the psychiatry clerkship identified a sense of perceived risk. The study highlighted the importance of utilizing evidence-based pedagogies, such as interactive reflective writing, to support the emotional development and professional identity formation of medical students. This suggests that incorporating reflective writing activities into medical education can provide students with a valuable opportunity to process their experiences, address emotional challenges, and enhance their professional growth [9]. Amini *et al.* conducted a study spanning from August 2012 to January 2013 involving 100 medical students undergoing training in the Paediatrics Department of Tabriz Children's Hospital. Initially, participants completed a questionnaire as a pre and post-test. Subsequently, during a workshop, they were instructed on the principles of reflection and learning domains. Each student was provided with a notebook titled What I Have Learned to record their daily reflections, categorized into three aspects: mirror, microscope, and binocular. After three months, the same questionnaires were administered as a pre & post-test. Statistical analysis was based on comparing the pre-test and post-test results along with the information gathered from the student's notebooks. Significantly different outcomes were observed for each question. Additionally, the study evaluated the pre-test and post-test results regarding knowledge of reflective writing, demonstrating a statistically significant improvement in post-test scores compared to pre-test scores. Reflective practice holds significant importance within teacher education and professional development initiatives. Engaging students in reflective practice is crucial for enhancing learning outcomes. Integrating reflection into learning processes cultivates clinical thinking skills and improves decision-making abilities. Particularly in today era of modern medical education, reflective practice plays a pivotal role. When students actively engage in reflection, they evaluate their work against established standards, analyze its effectiveness, and strategize for improvement [10]. We also compare between reflection writing in group as well as individually. Reflection in group is very effective as compare to writing reflection individually.

## Conclusion:

Reflective practice enhances the self-monitoring of students' learning processes and this contributes to their holistic and professional growth. Individual reflection leads to better academic performance compared to group reflection. This suggests that solitary reflection allows for deeper internalization and synthesis of material. Incorporating reflective writing activities into medical education provides valuable opportunities for students to process experiences and addresses emotional challenges and enhances professional growth.

## Figures and Tables

**Table 1:** Sample size distribution of Pre-test & Post-test

**Test**	**Sample size (n)**
Pre-Test	125
Post-Test	125

**Table 2:** Showing the comparison of Pre-test & Post-test

**Test**	**N**	**Mean**	**Std. Deviation**	**95% confidence interval of mean**		**Mean difference**	**t-test value**	**p-value**
				**Lower**	**Upper**			
Pre-test	125	3.86	0.86	3.7	4.01	-3.72	36.93	<0.001
Post-test	125	7.58	1.01	7.4	7.75			

**Table 3:** Showing the comparison between individually they wrote reflection & in group they wrote reflection

**Reflection**	**N**	**Mean**	**Std. Deviation**	**95% confidence interval of mean**		**Mean difference**	**t-test value**	**p-value**
				**Lower**	**Upper**			
Individually	62	38.05	4.41	36.93	39.17	10.59	15.21	<0.001
In Group	62	27.45	3.93	26.45	28.45			

**Table 4:** Showing the comparison between In Group they wrote reflection & individually they wrote reflection

**Reflection**	**N**	**Mean**	**Std. Deviation**	**95% confidence interval of mean**		**Mean difference**	**t-test value**	**p-value**
				**Lower**	**Upper**			
In Group	62	27.03	2.87	26.31	27.76	-11.19	15.44	<0.001
Individually	62	38.22	4.64	37.05	39.39			
